# Superficial Siderosis of the Central Nervous System Induced by a Single-Episode of Traumatic Subarachnoid Hemorrhage: A Study Using MRI-Enhanced Gradient Echo T2 Star-Weighted Angiography

**DOI:** 10.1371/journal.pone.0116632

**Published:** 2015-02-03

**Authors:** Hongwei Zhao, Jin Wang, Zhonglie Lu, Qingjie Wu, Haijuan Lv, Hu Liu, Xiangyang Gong

**Affiliations:** 1 Department of Radiology, Second Affiliated Hospital of Jiaxing University, Jiaxing, China; 2 Department of Radiology, Sir Run Run Shaw Hospital, Zhejiang University School of Medicine, Hangzhou, China; 3 Department of Neurology, Sir Run Run Shaw Hospital, Zhejiang University School of Medicine, Hangzhou, China; 4 Department of Radiology, Zhejiang Provincial People's Hospital, Hangzhou, China

## Abstract

The purpose of this study was to examine whether a single episode of traumatic subarachnoid hemorrhage (tSAH) could cause superficial siderosis of the central nervous system (SS-CNS).This study was approved by the local ethics committee. Thirty-two patients with a history of a single episode of tSAH were enrolled in the study. An episode of tSAH was confirmed in patients based on a CT scan or a lumbar puncture, and a follow-up examination was conducted at least six weeks after the brain trauma. A follow-up MRI examination was performed, using enhanced gradient echo T2 star-weighted angiography (ESWAN) to detect hemosiderin deposition on the cortical surface. The extent to which hemosiderin deposition was associated with several clinical factors was investigated. Various degrees of hemosiderin deposition were detected in 31 of 32 (96.9%) single-episode tSAH patients. Analysis of contingency tables revealed an association between the regions of subarachnoid bleeding based on CT images and the regions of hemosiderin deposition based on ESWAN images (χ^2^ = 17.73, P<0.05). SS-CNS was determined to be a common consequence after a single episode of tSAH. The extent of hemosiderin deposition is closely correlated with the initial bleeding sites and bleeding volume.

## Introduction

Superficial siderosis of the central nervous system (SS-CNS) is an uncommon condition that is due to the deposition of hemosiderin in the subpial layers of the brain, the spinal cord and certain cranial nerves. The classical clinic presentation of superficial siderosis is a triad of sensorineural hearing loss, progressive cerebellar ataxia and pyramidal signs [1,2]. A review of 270 published cases revealed that only 39% of patients presented with the classic triad; other symptoms included dementia, urinary and bowel problems, headache, anosmia, diplopia, ageusia and cranial-nerve palsies [3]. The pathogenesis of SS-CNS remains incompletely understood. At present, it is generally accepted that SS-CNS is due to small, chronic, repeated and often asymptomatic subarachnoid hemorrhages (SAHs) with various causes and that it typically occurs years after the initial bleeding. The most common causes of SAH that lead to SS-CNS include CNS tumors, head and neck trauma, CNS operations, and cerebral cavernous malformations [1,4–7]. A recent study presented a "localized"-type SS, apart from the “classic”-type SS. In localized-type SS, hemosiderin deposition is usually located in the cerebral cortex [8]. It has been determined that 35% to 54% of SS-CNS cases are caused by an idiopathic SAH originating from an occult bleeding source [1,3].

Prior to the advent of MR imaging, a diagnosis of SS-CNS was verified via autopsy or surgical exploration. With the widespread use of MRI, which has become the primary neuroimaging technique used to diagnose SS-CNS *in vivo* [4,9], SS-CNS is detected more frequently than in the past. MR imaging and postmortem correlation studies have demonstrated that hemosiderin deposition around the brain, the brain stem and the spinal cord characteristically display hypo-intensity on T2-weighted (T2WI) and gradient echo T2-weighted MR imaging (GRE T2*WI) [5,6,10,11]. Susceptibility-weighted imaging (SWI) is a new MR sequence that exhibits extreme sensitivity to blood-degradation products, such as iron and hemosiderin [12–14]. Deposition of hemosiderin on SWI presents as more thick and disseminated and with better resolution than T2WI and GRE T2*WI [6,7,15,16]. Enhanced gradient echo T2 star-weighted angiography (ESWAN), an SWI-like sequence, combines a unique 3D T2*-based multi-echo acquisition with a special reconstruction algorithm. It generates several echoes, which are read out at different TE times, and then combins them as a weighted average, compiling the magnetic signature of a wide range of tissues with varying degrees of T2* contrast [17]. A recent study revealed that ESWAN yields better resolution of lesions in patients exhibiting superficial siderosis and SAH and is equivalent or superior to GRE T2*WI for the diagnosis of various cerebral hemorrhagic lesions [18].

SAH is a common complication following traumatic brain injury (TBI). The incidence of traumatic subarachnoid hemorrhage (tSAH) ranges from 33% to 61% in TBI patients [19,20], and previous studies have reported disproportionately few tSAH cases that lead to SS-CNS [3]. To our knowledge, the question of whether a simple, single episode of tSAH can induce SS-CNS has not been answered in the literature. In the present study, our aims were (1) to determine whether SS-CNS can be induced by a single episode of tSAH and to assess its incidence, using a combination of high-field MRI units and an ESWAN sequence in a cohort of tSAH patients; (2) to evaluate the relationship between distribution and volume of tSAH and the consequent deposition of hemosiderin; and (3) to investigate the clinical factors that affect the extent of hemosiderin deposition.

## Materials and Methods

### Ethics statement

This study was approved by the Ethics Committee of the Second Affiliated Hospital of Jiaxing University and Sir Run Run Shaw Affiliated Hospital of Zhejiang University. All participants gave written informed consent prior to the MR examination. The consent form was signed by a parent or guardian if the patient was under 18 years of age. We acquired the patients’ written consent to publish their CT and MRI images.

### Subjects

A prospective group of patients experiencing tSAH was formed between January 2009 and October 2012 in two hospitals. Patients enrolled in the study met the following criteria: (1) tSAH was confirmed based a computed tomography (CT) scan or a lumbar puncture at the acute stage; (2) patients recovered well following conservative treatment without any invasive operation; (3) patients exhibited no history of previous craniotomy or central nervous system disease; and (4) patients returned to our hospitals 6 w or later after the first episode of head injury and received follow-up CT scans and MR examinations.

Forty-five patients injured in traffic accidents were initially enrolled in the study. We excluded 3 patients who used pacemakers, as such devices are hazardous in MR examinations; 5 patients were excluded due to unsatisfactory MR images; and 5 patients were excluded due to a lack of follow-up. Ultimately, 32 patients met the criteria of the study and completed the follow-up examinations. Of these, 22 were men and 10 were women, and the patients ranged in age from 8 to 87 years (mean age: 49.0±16.3 years). We also enrolled 25 healthy volunteers (15 men and 9 women; age range: 23 to 75 years; mean age: 48.2±15.5 years) as controls. There was no significant difference in age or gender between the patient group and the control group.

### Radiological examination

Of the 32 patients, 30 were diagnosed with tSAH based on a simple CT scan during the acute stage upon arrival at the emergency rooms of our hospitals. Two patients who tested negative on the CT examination but were strongly suspected to have tSAH were diagnosed based on a lumbar puncture and CSF examination. All patients completed their first CT scan within 6h of their head injury. The 16 slice scanners (Siemens Emotion & Sensation 16, Erlangen, Germany) used in the study were set at the following parameters: 120–130 kV, 240–285 mA, 9.6 mm slice thickness, 16 slices, no contrast enhancement. All patients underwent a follow-up CT scan using the same instrument.

Two high field MR scanners (1.5T Twin Speed Excite and 3T Signa HDxt, General Electric Medical Systems, Milwaukee, WI, USA), each containing an eight-channel phased-array head coil were used in the study. Twenty-four patients in the tSAH group and 25 healthy volunteers were examined using the 1.5T scanner, and 8 tSAH patients were examined using the 3.0T scanner. Following conventional Spin Echo sequence T1 and T2 weighted imaging, an ESWAN sequence was performed. The sequence was acquired from all 32 patients experiencing tSAH using a 3D-enhanced T2 star-weighted contrast flow-compensated system with a null gradient moment in all 3 orthogonal directions and a multiple-echo (11 different TEs) gradient sequence set at the following parameters: TR/TE/flip angle/band width/matrix size = 77 ms/10 ms-66.1 ms/30°/62.5Hz/; pixels/384×256 (1.5T); TR/TE/flip angle/band width/matrix size = 55.2 ms/5.5 ms-61.6 ms/20°/31.25Hz/; pixels = 416×320 (3.0T); and slice thickness = 2.0 mm, slice spacing = 0 mm, FOV = 23 cm×23 cm. All scans were oriented parallel to the anterior–posterior commissural (AC–PC) line, and 56–64 axial slices were obtained, covering the entire brain. The total acquisition time was 6 to 8 min, depending on the spatial ratio and the number of sections. The ESWAN source images were post-processed using FuncTool software (GE Healthcare) on an Advantage Windows (ADW) 4.3 workstation (Sun Microsystems, Santa Clara, CA, USA).

### CT and MR image analysis

**Figure 1:**
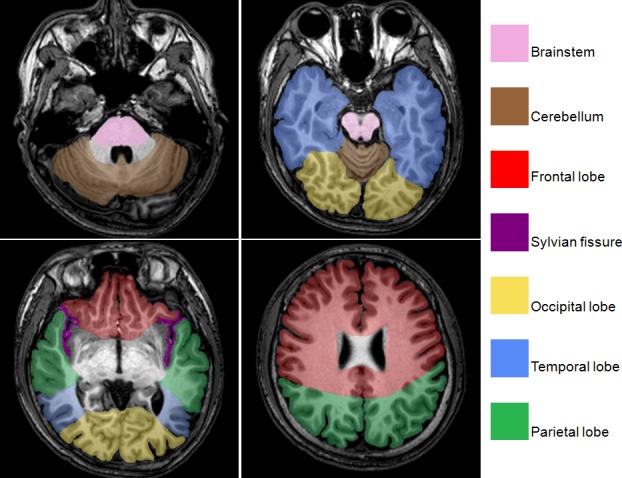
An MRI performed on a healthy subject using spin-echo T1-weighted imaging. We obtained axial slices of the normal brain. The anatomical structures of the subarachnoid space were labeled according to the following method. The subarachnoid area of each cerebral hemisphere was divided into 5 regions (frontal lobe, temporal lobe, occipital lobe, parietal lobe, and Sylvian fissure), and the subarachnoid areas of the cerebellum and the brainstem were divided into two relatively independent regions. Then, the number of regions, ranging from 0 to 12, of the subarachnoid area of the entire brain, excluding the ventricular system, was determined.

The distribution area of tSAH in each patient was divided into 12 regions according to the modified Imaizumi method [11]. The regions were the left and right frontal, temporal, occipital, parietal and Sylvian fissure areas, as well as the cerebellum and brainstem areas, excluding the ventricular system. We counted and summed the regions displaying SAH to determine the severity and dissemination of tSAH (Fig. 1).

**Figure 2:**
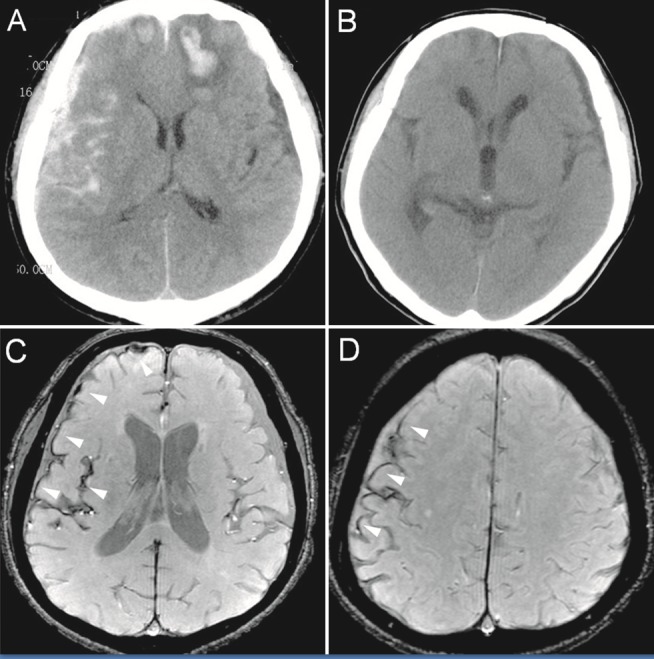
CT and enhanced gradient echo T2 star-weighted angiography (ESWAN) images of the brain of a 54-year-old man who experienced a traumatic brain injury. An axial head CT image displays right frontotemporal SAH (Fisher grade 4) with bilateral frontal contusions and intracerebral hematoma (A). A follow-up CT image 26 weeks after the brain injury indicates that the hemorrhages were completely resolved and the lateral ventricles were mildly enlarged (B). A follow-up MRI (1.5T) image was obtained 26 weeks following the head injury (C,D). The axial ESWAN image displays a rim of hypointensity (*arrowheads*), with hemosiderin deposits forming along the cerebral convexity (C, D).

We adopted the widely used Fisher classification of SAH [21] and applied modifications for tSAH. Grade 1 represents a positive result based on a lumbar puncture but with no blood detected on the CT scan; grade 2 represents a diffuse or thin-layered deposition of blood in the subarachnoid space without clots or vertical layers of blood 1 mm or greater in thickness; grade 3 represents localized clots and/or vertical layers of blood 1 mm or greater in thickness (thickness <3 mm); and we modified grade 4 by including diffuse subarachnoid blood (thickness >3 mm) regardless of intraventricular hemorrhage. The Fisher Grade for tSAH was based on the consensus findings of two experienced neuroradiologists using the initial cranial CT image (Fig. 2A).

SS-CNS was evaluated via the follow-up MR ESWAN images. SS-CNS was defined as a low signal-intensity rim along the surface of the brain on three or more cross-sections, excluding the cortical vessels, which also displayed low signal intensity in T2*WI and ESWAN images (Fig. 2C, 2D). We divided the brain surface into 12 regions using the method described above and recorded the number of areas displaying deposition.

Two experienced neuroradiologists, blinded to the clinical histories of the subjects, independently evaluated the 57 sets of images, including those of the 32 tSAH patients and the 25 controls.

After the two neuroradiologists finished reviewing all of the data initially, they discussed their results and came to an agreement.

### Statistical analysis

Weighted k values of Kappa statistics were calculated to estimate inter-observer agreement with respect to the presence or absence of SS-CNS based on the ESWAN images. Weighted k values from 0.00 to 0.20 represented poor agreement; 0.21 to 0.40 represented fair agreement; 0.41 to 0.60 represented moderate agreement; 0.61 to 0.80 represented good agreement; and 0.81 to 1.00 represented excellent agreement.

Correlations between regions displaying tSAH distribution and those displaying SS-CNS deposition were analyzed using contingency tables. Student’s *t*-tests and Fisher's exact tests were conducted to analyze the effect of each clinical factor on hemosiderin deposition.

Statistical analyses were performed using SPSS (Statistical Package for the Social Sciences) for Windows, version 13.0 (SPSS, Chicago, IL, USA). A statistically significant difference was defined as a *p* value of ≤ 0.05.

## Results

After the Fisher scale was applied to the initial cranial CT images, two patients were classified as grade 1, 16 patients as grade 2, 5 patients as grade 3, and 9 patients as grade 4. Among the 30 patients who displayed SAH as determined by CT image (grades 2–4), the distribution of SAH was 29.1% (25/86) in the frontal lobe, 14.0% (12/86) in the temporal lobe, 1.2% (1/86) in the occipital lobe, 15.1% (13/86) in the parietal lobe, 29.1% (25/86) in the Sylvian fissure, 1.2% (1/86) in the cerebellum and 10.5% (9/86) in the brainstem area.

The presence or absence of SS-CNS was evaluated independently by two neuroradiologists based on the ESWAN images of both the tSAH and control groups. The Kappa statistic of inter-observer agreement for the estimated positive scores of SS-CNS was 0.859 (p< 0.001), indicating strong agreement between the two evaluators regarding the presence or absence of SS-CNS.

**Figure 3:**
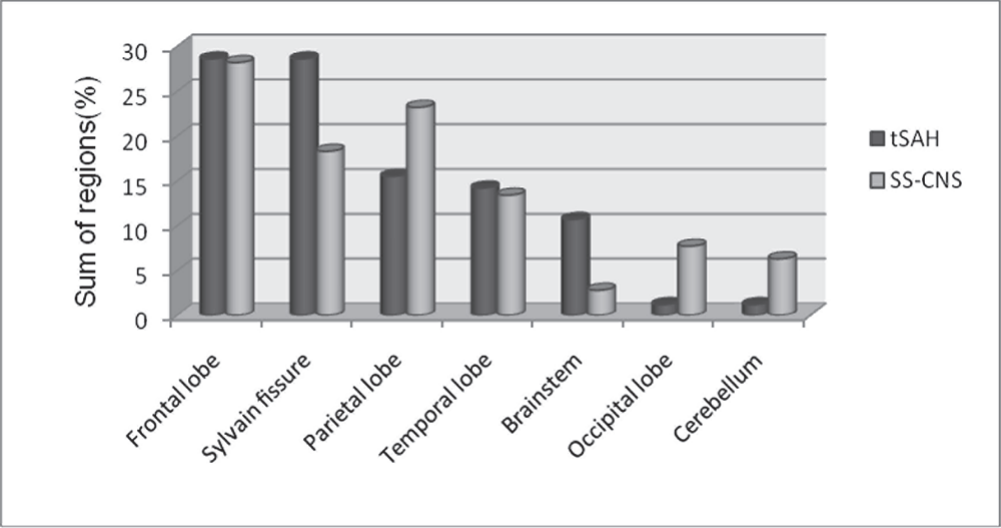
Chi-square contingency table analysis reveals a correlation between the number of regions exhibiting tSAH (%) based on CT images and the number of regions exhibiting SS-CNS (%) based on ESWAN images in 31 patients (*χ* ^*2*^ = 17.73, *P*<0.05).

In the tSAH group, SS-CNS was detected in 31 of 32 patients (96.9%) based on the ESWAN images. Only one patient in the tSAH group exhibited no evidence of SS-CNS; the Fisher grade for this patient was 2. All of the 25 healthy volunteers were SS-CNS negative on ESWAN images. For the thirty-one patients with SS-CNS, the median time interval between injury and MRI scan was 26 weeks (range 6–177 weeks), and the distribution included time intervals of 6–10 weeks (n = 5), 11–20 weeks (n = 7), 21–30 weeks (n = 6), 31–40 weeks (n = 6) and >40 weeks (n = 7). The total number of regions containing hemosiderin deposits among the tSAH patients was 142 (mean: 4.43 per patient). The distribution of the regions containing deposits consisted of 28.2% in the frontal lobe (40/142), 23.2% in the temporal lobe (33/142), 7.7% in the occipital lobe (11/142), 13.4% in the parietal lobe (19/142), 18.3% in the Sylvian fissure (26/142), 6.3% in the cerebellum (9/142) and 2.8% in the brainstem area (4/142). Chi-square contingency table analysis revealed a significant correlation between the distribution of subarachnoid bleeding based on the CT images and the distribution of SS-CNS deposition based on the ESWAN images (χ^2^ = 17.73, P<0.05) (Fig. 3).

**Figure 4:**
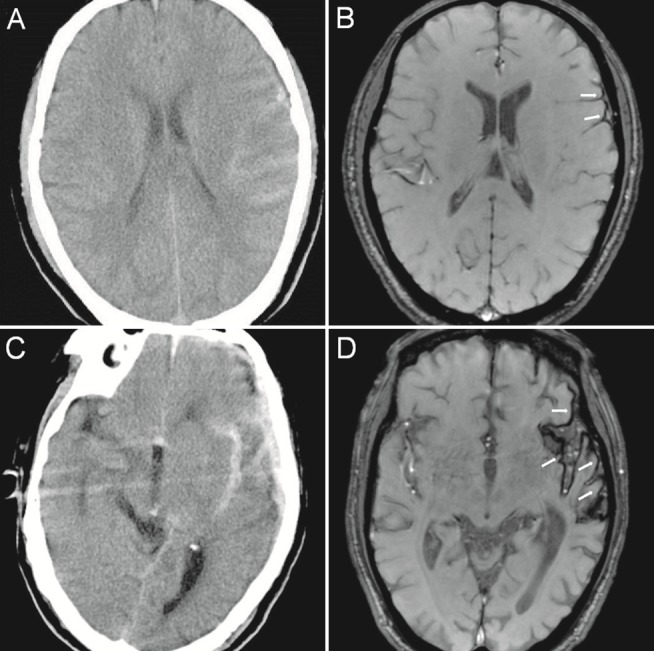
CT and ESWAN images of the brain of a 50-year-old man (A, B) and a 59-year-old man (C, D). The Fisher grades of the SAH based on the CT images were grade 2 (A) and grade 4 (C), respectively. Follow-up MR (1.5T) images were obtained at 34 weeks (B) and 177 weeks (D) after the head injury. The *arrows* denote areas of SS-CNS, which is indicated by a rim of hypointensity on the surface of the brain. Note the extent of deposition in each case. The CT and ESWAN images display a degree of correlation, patients with a higher Fisher grade exhibit more regions containing SS-CNS deposits.

The number of regions containing SS-CNS deposits was 8 among the 2 patients with a Fisher grade of 1, 46 among the 15 patients with a Fisher grade of 2 (mean: 3.1 per patient), 22 among the 5 patients with a Fisher grade of 3 (mean: 4.4 per patient) and 66 among the 9 patients with a Fisher grade of 4 (mean: 7.3 per patient). There appeared to be some correlation between the Fisher grade based on the CT images and the mean number of SS-CNS regions per patient based on the ESWAN images. In patients with a higher Fisher grade (grades 2–4), more regions containing SS-CNS deposits were detected (Fig. 4).

The factors affecting the extent of hemosiderin deposition, based on the ESWAN sequence images, were analyzed using univariate statistics (Table 1). The results indicate that a higher Fisher grade (≥3), based on the initial CT images, is significantly associated with the spread of hemosiderin deposits. Neither age, gender nor the interval between the initial CT scan and the ESWAN scan affected the extent of SS-CNS deposition.

**Table 1:** Univariate analyses of factors associated with extent of SS-CNS deposition based on ESWAN in 31 patients.

	SS-CNS regions ≤4	SS-CNS regions ≥5	P
No. of cases	16	15	-
Age (years)	48.6±14.4	51.1±17.8	0.679^*^
≥50 years/<50 years	9/7	9/6	1.000
Sex (male/female)	9/7	12/3	0.252
Fisher grade 3 (±)	3/13^†^	11/4^‡^	0.004^**^
Interval (CT-MR)<26W	8/8	7/8	1.000

SS, superficial siderosis; ESWAN, enhanced gradient echo T2 star-weighted angiography.

*Student’s t-test, t = −0.434, P>0.05.

**Fisher's exact test, P<0.05.

†3 (3 patients classified as grade 3), 13 (11 patients classified as grade 2 and 2 patients as grade 1).

‡ 11 (2 patients classified as grade 3and 9 patients as grade 4), 4 (4 patients classified as grade 2).

## Discussion

Iwanowski and Olszewski reproduced superficial siderosis in dogs after repeated subarachnoid injections of blood or iron dextran [22]. In another animal study, Koeppen and colleagues experimentally induced SS-CNS in a rabbit model after weekly intracisternal injections of autologous red blood cells for 6 months [23]. Koeppen *et al*. reported that chronic or intermittent extravasations of blood into the subarachnoid space and the dissemination of heme in the circulating cerebrospinal fluid are the only established causes of SS-CNS. It has also been reported that siderosis does not occur in humans after a single episode of SAH and that red cells can be cleared from the CSF over time (2). This hypothesis was universally accepted because more than 60% of the detected cases exhibited preexisting bleeding sources. Progression of SS-CNS has been halted to some degree after treatment of the source of SAH [24,25].

However, there has yet to be any clinical or experimental study that discusses the relationship between a single episode of SAH and SS-CNS. Previous studies of patients with ruptured cerebral aneurysms have confirmed that aneurysmal SAH will lead to SS-CNS [11]. Kakeda [26] examined 47 patients who experienced subdural hematoma (SDH) and 36 patients who experienced aneurysmal SAH. SS-CNS was detected in 13 patients (27.7%) in the SDH group. It is well-known that, SAH caused by an aneurysm rupture is not truly a single episode of SAH because repeated occult bleeding may occur prior to the aneurysm rupture, and subsequent craniotomy and clipping of the intracranial aneurysm increase the probability of developing SS-CNS. In this study, we found for the first time, to our knowledge that a single episode of tSAH will induce SS-CNS in almost all patients, even those with a very minor SAH. Our study supports the theory of pathogenesis of SS-CNS.

In this study, SS-CNS preferentially accumulated in areas where tSAH was distributed, as observed in initial cranial CT images. Depositions in supratentorial regions accounted for 90.8% of the total depositions, and subtentorial depositions accounted for the remaining 9.2%. A close relationship was found the between regions of SS-CNS deposits and the initial distribution of tSAH. These findings are quite different from those in patients exhibiting “classic” SS-CNS. Our study also indicated that SS-CNS dissemination appears to be correlated with the severity of tSAH. Patients with higher Fisher grades and a greater extent of bleeding exhibited a broader distribution a greater extent of hemosiderin deposition. The combination of previous evidence and our results indicates that SS-CNS is indeed a common consequence of many types of SAH. We prefer to regard SS-CNS as a pathological change rather than a distinct entity. The clinical presentations of patients with SS-CNS include a spectrum ranging from asymptomatic to the classic triad, depending on the deposition site, the distribution, and the severity and course of the disease as well as individual variables.

Various therapeutic methods have been proposed to treat SS-CNS, including steroid therapy [27], antioxidants and radical scavengers [28] and oral deferiprone [29]. However, none of these methods has been proven to reverse the damaging effects of hemosiderin deposition in the brain. Our study indicates that a single episode of tSAH is a substantial risk factor for the development of SS-CNS. We propose that hemosiderin deposition on the brain surface (SS-CNS) should be considered as a late complication of tSAH. An adjustment of the management strategy for tSAH in the acute stage may be worthwhile.

Our study has several limitations. First, we do not possess a histological standard by which we could determine the accuracy of our diagnoses. Second, the patient sample was too small. To draw meaningful conclusions, a multicenter study involving a larger number of patients is required. Third, although all patients enrolled in the study had no history of previous craniotomy or central nervous system disease, we could not fully exclude the possibility of preexisting SS in some patients because we did not perform an MRI examination before or immediately after brain injury. Fourth, clinical symptoms, neuropsychological outcomes and prognoses were not evaluated. Finally, the differences in MRI parameters used and the timing of the scans after tSAH should be considered in future studies.

In conclusion, based on our observations of 32 patients and a combination of high-field MR and ESWAN sequences, we found that a single episode of tSAH can induce SS-CNS in a majority of tSAH patients. The extent of hemosiderin deposition is closely correlated with the initial bleeding sites and bleeding volume. Patients’ age and gender and the interval between the initial CT and ESWAN scans did not affect the extent of SS-CNS deposition.
